# Virtual Reality as an Affirmative Spin-Off to Laparoscopic Training: An Updated Review

**DOI:** 10.7759/cureus.17239

**Published:** 2021-08-17

**Authors:** Ketan Kantamaneni, Krishi Jalla, Mahvish Renzu, Rahul Jena, Amudhan Kannan, Ruchi Jain, Suchitra Muralidharan, Vijaya lakshmi Yanamala, Zainab Zubair, Jerry Lorren Dominic, Myat Win, Anjli Tara, Sheila W Ruo, Michael Alfonso

**Affiliations:** 1 General Surgery, California Institute of Behavioral Neurosciences & Psychology, Fairfield, USA; 2 General Surgery, Dr. Pinnamaneni Siddhartha Institute of Medical Sciences and Research Foundation, Gannavaram, IND; 3 Internal Medicine, California Institute of Behavioral Neurosciences & Psychology, Fairfield, USA; 4 Internal medicine, California Institute of Behavioral Neurosciences & Psychology, Fairfield, USA; 5 Diagnostic Radiology, California Institute of Behavioral Neurosciences & Psychology, Fairfield, USA; 6 Dermatology, California Institute of Behavioural Neurosciences & Psychology, Fairfield, USA; 7 General Surgery, Stony Brook Medicine/Southampton Hospital, New York, USA; 8 General Surgery and Orthopaedic Surgery, Cornerstone Regional Hospital/South Texas Health System, Edinburg, Texas, USA; 9 General Surgery, Vinayaka Mission's Kirupananda Variyar Medical College, Salem, IND; 10 General Surgery, Nottingham University Hospitals NHS Trust, Nottingham, GBR; 11 General Surgery, Liaquat University of Medical and Health Sciences, Jamshoro, PAK; 12 School of Medicine, Universidad del Rosario, Bogota, COL; 13 Medicine, California Institute of Behavioral Neurosciences & Psychology, Fairfield, USA

**Keywords:** laparoscopy, virtual reality, haptics, surgery, simulator, surgeon, box trainer, technology, outcomes

## Abstract

Latest advancements in science lead to drastic improvements in patient health care. Techniques and technology evolved in surgery over the years have resulted in the improvement of patient outcomes by leaps and bounds. Open surgeries previously done for procedures like appendectomy and cholecystectomy evolved into laparoscopic minimally invasive procedures. Such procedures pose few challenges to the surgeons, like lack of tissue feedback and fulcrum effect of the abdominal wall. But training surgeons for such an advanced skill is still following conventional methods. These procedures can be effectively trained using Virtual Reality (VR), which can simulate operations outside the operating room (OR). To maximize the outcomes of VR training, knowledge on various strategies affecting the skills acquisition and retention in VR training is essential. This review collected information from PubMed, EMBASE, Cochrane Library (CENTRAL) databases. Data from the previous ten years are included in the review. This included documents, clinical trials, meta-analysis, randomized controlled trials, reviews, systematic reviews, letters to editors, and grey literature. After an advanced Medical Subject Headings (MeSH) search, we got 59,532 results, and after the application of filters, 189 results showed up. Out of these, studies that were not exclusively relevant to the use of VR in laparoscopic surgery were manually excluded, and a total of 35 articles were included in the study. VR is found to be an excellent training modality with promising outcomes. It helps the surgeons perform the surgery accurately at a faster pace and improves confidence and multitasking ability in OR. Instructor feedback from mentors and deliberate practice of trainees, and early introduction of haptics in VR resulted in the most effective outcomes of the VR training. Box trainers are also compared with VR trainers as they are the cheaper modalities of training. However, this area needs more research to conclude if box trainers can act as a cheaper alternative to VR training providing similar outcomes.

## Introduction and background

Virtual Reality (VR) is a simulated system generated by a computer to provide an experience similar to or completely different from the real world. It has many applications in gaming, military training, and astronaut training, among others. Many studies have been published to determine the effectiveness of VR in the surgical training field [1]. Minimally invasive surgery gained widespread importance in recent times. Laparoscopy requires unique skills which are non-transferable from open surgery [2]. For this type of surgery, psychomotor and hand-eye coordination are essential. However, it is widely accepted that training for laparoscopic surgery can be done in virtual laboratories before performing in the OR as it is a safe, controlled, standardized, and repeatable environment [2, 3].

The traditional surgical training system advocating "see one, do one, teach one" is slowly losing importance. VR training ensures safer health care systems and a reduction in the number of surgical errors [4]. Virtual reality simulators used in laparoscopic training include Minimally Invasive Surgical Trainer Virtual Reality (MIST VR), LapSim®, SimSurgery, Lap Mentor^TM^, Sinergia [5]. "Out of OR" training is possible with the use of VR for laparoscopic procedures like appendectomy, cholecystectomy, etc., which is found to make a profound difference in their performances in OR and shortens their learning curve [6, 7]. During their laparoscopic training, the major challenges faced by trainee surgeons are their limited time available for training, fulcrum effect of the abdominal wall, and lack of haptic feedback. VR laparoscopic training is found to be an effective method to address all these challenges and enables them to learn the skills in a risk-free environment [8, 9]. It has been a long while since the introduction of VR systems in training laparoscopic surgeons. However, there is a scarcity of long-term review literature targeting the efficacy of VR in laparoscopic training and the variety of factors playing a role in improving or depreciating the performance of laparoscopic surgeons in VR. Hence this standard review article aims to bridge the literature gap by reviewing the articles of the past ten years related to the use of VR in laparoscopic training.

## Review

Methods

A traditional review was carried out until June 1, 2021. Databases used for the search were MEDLINE (PubMed), EMBASE (Ovid), Cochrane Library (CENTRAL). The Medical Subject Headings (MeSH) terms used for the search in PubMed are MeSH “Virtual Reality” AND MeSH “Laparoscopy” AND MeSH “Surgery” AND MeSH “Training.” Articles of the previous ten years and only those in English, including books and documents, clinical trials, meta-analysis, randomized controlled trials (RCT), reviews, systematic reviews, letters to editors, were taken into study. Articles that are not exclusively related to ‘laparoscopic surgical training,’ including the use of VR in other surgical and medical training, augmented and mixed reality, and VR for rehabilitation, were excluded. Articles on other forms of training were also excluded.

Result

After an advanced MeSH search, we got 59,532 results, and after the application of filters, 189 results showed up. Out of these, papers that were not exclusively relevant to the use of VR in laparoscopic surgery were manually excluded, and a total of 35 papers were included in the study.

Discussion

Effect of VR on Mental Workload in Novice Laparoscopic Surgeons

Surgery is one of the demanding branches which puts its trainees to extreme mental and physical workload. A prospective controlled simulation study was conducted by Barré et al. from January 2018 to September 2018 taking ten residents in surgery from the Institut Mutualiste Montsouris in Paris, who were novices in laparoscopic sleeve gastrectomy, using the VR training module designed by VirtualiSurg company on HTC Vive headset. The study showed that the mental workload measured with the National Aeronautics and Space Administration Task Load Index (NASA-TLX) score and physical workload measured with the Borg scale and the manikin discomfort test were significantly reduced in the VR training group compared to the control group, which didn't show much of a difference. Hence the authors advocated VR training as a method to reduce the physical and mental workload on surgeons in OR [10]. However, a study conducted by Frederiksen et al. showed that immersive VR training (with head-mounted displays) caused more cognitive load to trainees than conventional VR [9]. Hence the author advocated the use of immersive VR only after initial training on conventional VR.

Use of VR in Designing Curriculum-Based Training

**Figure 1:**
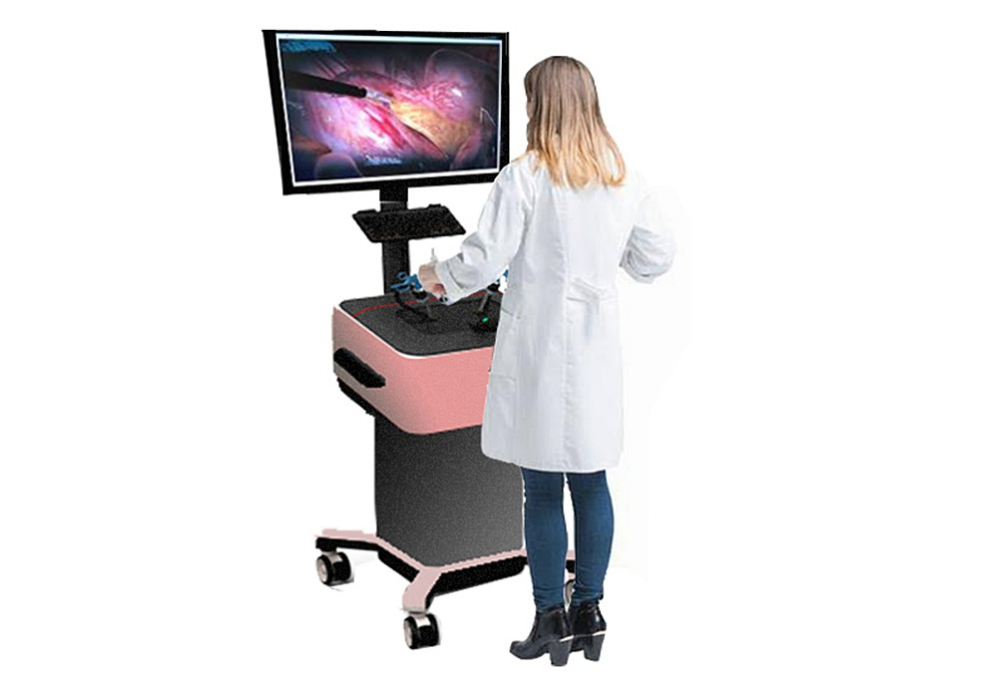
Virtual Reality (VR) trainer Original figure, made by the author KK

An RCT performed by Beyer-Berjot et al. involving 20 novice surgeons, seven intermediate surgeons, and six experienced surgeons using the Lap Mentor^TM^ showed that VR training improves surgeon's performance in specific tasks in laparoscopic surgery in metrics like time, the number of movements, and path length excluding the number of movements in the anastomosis module [6]. A study conducted by Gallagher et al. also showed that surgeons trained on Minimally Invasive Surgical Trainer Virtual Reality (MIST VR) computer program made accurate incisions under laparoscopy laboratory conditions, thus proving that VR training can help attain required psychomotor skills for laparoscopy [11]. RCT performed by Larsen et al. and Nagendran et al. also proved that skills learned in VR trainers can be successfully transferred to real operations and also a reduction in operating time [12, 13]. RCT conducted by Torkington et al. also proved the same [14]. Considering the ethical issues involved in using animals and cadavers for laparoscopic simulation, VR can act as an effective method to develop curriculum-based training taking advantage of its repeatability in a safe environment. VR trainer is shown in Figure 1.

Factors Affecting VR Laparoscopic Training Outcomes

It is important to understand the factors affecting skills acquisition and retention to develop a VR training program.

Instructor feedback:

An RCT was performed by Bjerrum et al. to assess skill retention after VR training after six months using LapSim for laparoscopic salpingectomy. The author noted that skills decay started after 6-18 months without practice. This study also showed that, however, instructor feedback during VR improved the efficiency of initial VR training, though it has no effect on skills retention [7]. Also, in a study conducted by Paschold et al., the trainees were divided into low performer group (LPG) and high performer group (HPG) based on their initial performance of procedural tasks on VR simulator. Then tailored verbal instructor feedback (VIF) was given only to LPG. After completion of training, a post-test (clip applying task) was conducted on LapSim VR trainer to both the groups. It was found that with VIF, LPG performance equalled HPG [15]. A similar study to assess the effect of instructor feedback on virtual reality was also conducted by Oestergaard et al., but the results have not been published by the time of completion of this study [16].

Multitask training:

Distraction can harm laparoscopic performance. One of such distractions is insufflator problems in the OR. An RCT conducted by Bongers et al. evaluated the use of VR training to improve multitasking in OR to overcome distraction. The intervention group was trained using both VR laparoscopic trainer and a laparoscopic insufflator trainer. Scenarios such as intra-abdominal pressure build-up problems, tube obstruction, gas supply problems were simulated and trained. The intervention group was successfully able to "task switch," and solve the insufflator problems and complete the surgery early in the post-test. This showed that two modules, when combined trained in VR, can enable multitasking [4].

Warmup before VR:

Several warmup strategies have emerged intending to improve laparoscopic performance. In a study conducted by Brönnimann et al., few participants were given hands-on warmup (playing table soccer), few were given cognitive warmup (playing tablet 3D game on iPad) and others were control group with no warmup. Then all of them were subjected to laparoscopy training in VR. The hands-on warmup group didn't show any performance improvement, whereas the iPad group showed improved performance in camera navigation but no significant improvement in hand-eye coordination and two-handed maneuvers [17].

Competitive training:

The competition had led to improved performance in sports. The same was tried with VR laparoscopic training. In an RCT conducted by Hashimoto et al., 20 surgical novices were randomized into competitive training (CT) group and control group to perform 10 VR laparoscopic cholecystectomies (LC). CT group were told that they were under competition to win a prize. Performance was assessed using the Objective Structured Assessment of Technical Skills Global Rating Scale (OSATS GRS) score. It was expected that the CT group would show significant improvement than the control group. But surprisingly, it was found that there was no significant difference in OSATS GRS score between the two groups; however, the CT group showed greater dexterity [18].

Deliberate practice:

Deliberate practice involves individuals repeatedly practicing on tasks and getting immediate feedback so that they can focus on training to overcome their weaknesses while also working on refining other aspects of performance. RCT conducted by Hashimoto et al. randomized 20 laparoscopic novices into deliberate practice (DP) group and control group. Both the groups were subjected to 10 VR sessions comprising a total of 20 VR laparoscopic cholecystectomies (LC) on the Lap Mentor^TM^ VR laparoscopic simulator, and their performances were continuously assessed using OSATS GRS score. The DP group was constantly given feedback on their weaknesses by the qualified observer after each session based on their scores and methods to improve them. Then they were subjected to 30 min of deliberate practice on Lap Mentor^TM^ VR trainer or LapSim® VR trainer to improve on their weaknesses while the control group spent 30 minutes watching Ted talks or reading journals. After these VR sessions, both the groups were subjected to LC on a cadaveric porcine model using real surgical instruments and rated on OSATS GRS score. DP group people scored significantly higher scores than the control group. The author also mentioned that DP could lead to improved performance to the level of an expert. However, lapses in practice can cause "arrested development" and premature plateauing of performance [19]. Similar findings were also observed in RCT conducted by Palter et al., further strengthening the evidence [20].

Skill transfer from other procedural training: 

Yang et al. conducted an RCT with surgical novices who were randomized into group 1 (trainees underwent appendectomy VR training and then cholecystectomy VR training) and group 2 (trainees underwent cholecystectomy VR training directly). Both the groups underwent basic training of five laparoscopic tasks (clipping and grasping, cutting, electrocautery, peg transfer, and pattern cutting) in VR and were then subjected to post-test on laparoscopic cholecystectomy (LC) on VR simulator. It was found that there was no improved performance on VR LC with prior practice of VR appendectomy. However, the movements of group 1 participants were more economical. Hence, this shows that though the participants may benefit from the transfer of motor skills, the procedures need to be trained separately [21].

Multimodality training:

In the RCT conducted by Kowalewski et al., participants were randomized into multimodality training (MMT) group and control group. Pretest (LC on the porcine liver) was performed for both groups. Then MMT group was trained for basic skills on box trainer for six hours and the procedure of laparoscopic cholecystectomy on VR trainer for another six hours. Then both these groups were subjected to a post-test on a VR trainer. Their performance was assessed using the Global Operative Assessment of Laparoscopic Skills (GOALS) score. MMT group scored significantly higher than the control group. It was also found that after MMT the expertise of junior residents matched that of senior residents, thus showing that MMT is beneficial to achieve better outcomes in VR training [22]. RCT conducted by Sumitani et al. also showed the same results [3]. Also, in the study conducted by Lesch et al., where candidates were randomized into a video training group and VR laparoscopic cholecystectomy training group, the post-training questionnaire showed that video training is easy to use than VR training. However, VR training showed improved confidence than video training. So the participants suggested that the trainees should get to read the steps of the procedure first; they should be trained with a video trainer and then with a VR trainer for best outcomes [23].

Paired team training:

When trainees are teamed up in pairs during training sessions, this leads to the exchange of knowledge, discussions, reduction of stress through intraoperative breaks. An interesting study conducted by Nickel et al. randomized surgical novices into group A (multimodality training alone), group B (multimodality training in pairs), group C (no training). Group B candidates got lesser repetitions at a trainer as they had to take turns to train at the same time as that provided to other groups. Post-test was conducted to all the groups on VR trainer and porcine cadaveric LC, and OSATS score was used for the assessment. However, the trial has not been completed by the time of publication of this study [24]. Hence the effect of team training on VR outcomes is still questionable.

Haptic feedback:

One of the crucial things that VR trainers lack is the haptic feedback of the tissue. Introducing haptic feedback into VR trainers can lead to the use of different senses of the surgeon and may further lead to better and faster performance. In the RCT conducted by Ström et al., 38 surgical residents were randomized into the early haptic (EH) group and late haptic (LH) group. EH group were trained in VR trainer with haptic feedback for one hour and then without haptics for another hour. LH group started training without haptics first and then with haptics. Then both groups were subjected to the BasIQ general cognitive ability test and Mental Rotation Test A (MRT-A). It was observed that early haptic group performance was significantly higher in manipulating and diathermy (MD) tasks and point diathermy (PD) tasks in the tests. Hence the early introduction of haptic feedback results in improved VR outcomes [25]. All the RCTs mentioned above are summarized in Table 1.

**Table 1:** Characteristics of the included studies RCT - Randomized Controlled Trial; CBC - Competency-Based Curriculum; VR - Virtual Reality; OSATS GRS - Objective Structured Assessment of Technical Skills Global Rating Scale; GOALS - Global Operative Assessment of Laparoscopic Skills; CT - Competition Training; DP - Deliberate Practice; LC - Laparoscopic Cholecystectomy; TIPS - Toolkit for Illustration of Procedures in Surgery; NASA-TLX - National Aeronautics and Space Administration Task Load Index; MIST VR - Minimally Invasive Surgical Trainer Virtual Reality; POP - pulsating organ perfusion

Author	Type of study	Purpose of study / Objective	Platform (s)	Assessment methods:	Results
Barré et al. [10]	RCT	Effect of VR training on mental & physical workload of surgeons	HTC Vive headset	NASA-TLX, Borg scale, and manikin discomfort test	Decreased mental&physical workload
Beyer-Berjot et al. [6]	RCT	Can virtual reality competency-based curriculum (CBC) enhance performance during real surgical procedures?	Lap Mentor^TM^	Metrics given by Lap Mentor^TM^	CBC reduced the learning curve during real colorectal resections.
Bjerrum et al. [7]	RCT	Effect of instructor feedback on long-term skill retention	LapSim® VR simulator	Metrics given by LapSim® VR simulator	Instructor feedback during proficiency-based laparoscopic simulator training didn't affect the long-term retention of skills.
Bongers et al. [4]	RCT	Can multitasking and task switching be trained in a virtual reality (VR) laparoscopic skills simulator?	SIMENDO VR simulator	5 point Likert scale, time taken measured by VR simulator	Multitask training in VR simulator enabled surgeons to solve secondary tasks quicker.
Brönnimann, MD et al. [17]	RCT	Effect of warmup strategies on VR performance	Lap Mentor^TM^	Metrics given by VR simulator	Warm-up strategies did not affect VR performance in laypersons.
Buescher et al. [8]	RCT	Effect of continuous motion parameter feedback on laparoscopic simulation training	Lap-X Hybrid laparoscopic simulator, LAP Mentor II	Metrics given by VR simulator	Continuous motion feedback improved laparoscopic skill enhancement significantly in several aspects.
Frederiksen et al. [9]	RCT	Effect of immersive VR (vs) conventional VR simulation on cognitive load and performance	Conventional & immersive laparoscopic simulation setup consisting of two Simball™ 4D joysticks	Secondary task reaction time as a measurement for cognitive load & simulator metrics for performance	Immersive VR simulation training resulted in a higher cognitive load and a poorer performance than conventional VR simulation training in laparoscopy.
Gallagher et al. [11]	RCT	Can VR training help in overcoming the "fulcrum effect"?	MIST VR & box trainer with an Olympus video CCD miniature camera	Endoscopic evaluation test	VR training lead to making accurate incisions & to overcome the "fulcrum effect"
Hashimoto et al. [18]	RCT	Effect of competition training on the development of laparoscopic surgical skills	Lap Mentor^TM^ VR	[OSATS GRS] score	CT lead to improved dexterity in laparoscopic surgery but didn't yield improved performance than that of standard training in novices
Hashimoto et al. [19]	RCT	Effect of deliberate practice on quality of laparoscopic surgical performance	Lap Mentor^TM^ VR	Global (GRS) and procedure-specific (PSRS) rating scales	DP lead to higher quality performance in VR LC than standard training alone
Kowalewski et al. [22]	RCT	Evaluation of benefits of a combined multi-modality training program for surgical residents	Porcine liver, box trainers including POP trainers, Lap Mentor 2*	GOALS SCORE	Structured multi-modality training benefitted novices to overcome the initial learning curve in laparoscopy and to decrease operation time for LC.
Larsen et al. [12]	RCT	Effect of virtual reality training on an actual laparoscopic operation	LapSim® Gyn v 3.0.1	Objective structured assessment of laparoscopic salpingectomy score	VR training resulted in improvement of operating skills during actual procedure and learning curve was also shorter and operating time was halved
Lesch et al. [23]	RCT	Effect of VR training in teaching laparoscopic surgical techniques to medical students when compared to passive learning tools like videos.	Toolkit for Illustration of Procedures in Surgery (TIPS), WISE-MD videos	Appendectomy post quiz, cholecystectomy post quiz	TIPS simulation appeared to be a useful adjunct to the learning environment, also improved student confidence. However, students found WISE- MD videos easy to use
Nickel et al. [24]	RCT	Assessing the differences in laparoscopy training for laparoscopic beginners by using a single workplace to train one (vs) two trainees simultaneously.	Online learning modules, box trainer, VR trainer, Porcine cadaver in the POP trainer	(OSATS)SCORE,(GOALS) SCORE, VR METRICS	Trial was not completed by the time this RCT was published
Oestergaard et al. [16]	RCT	Effect of instructor feedback versus no instructor feedback on performance in a laparoscopic virtual reality simulator	LapSim®, Simball™ 4D Joystick	Metrics given by VR simulator	Trial was not completed by the time this RCT was published
Palter et al. [20]	RCT	Assess the effect of individualized deliberate practise on a virtual reality (VR) simulator on technical performance in the operating room.	Laparoscopic cholecystectomy on the patient, LapSim® VR simulator	(OSATS) score, the modified OSATS and Likert scale	Residents in the deliberate practice group showed superior technical performance in the operating room.
Paschold et al. [15]	RCT	The effect of verbal instructor feedback on virtual-reality laparoscopic (VRl) training	LapSim® VR simulator	Metrics provided by VR simulator	Verbal feedback to low performing group of surgeons resulted in significant improvement in their performance.
Ström et al. [25]	RCT	Assess the effect of early introduction of haptics on simulator performance	Procedicus abdomen simulator	BasIQ general cognitive ability test, revised Vanderberg and Kuse mental rotation test, form A (MRT-A), Borg CR-10 scale.	There was no significant difference between the two groups (early haptic vs late haptic) in visual-spatial ability, however early haptic feedback group significantly improved on manipulate & diathermy task after two hours of training
Torkington et al. [14]	RCT	Assessing the transfer of skills achieved by the use of virtual reality simulators to real tasks	Box trainer, MIST VR simulator	Scores generated by Imperial College Surgical Assessment Device	No difference is observed in the time taken to complete the procedure after training in VR. However greater economy of movement is observed in VR trained group
Yang et al. [21]	RCT	Assessment of transferability of surgical skills between two laparoscopic abdominal procedures using the virtual reality simulator	Lap Mentor^TM^	Metrics provided by VR simulator	Previous VR appendectomy training didn't improve time and safety parameters in VR cholecystectomy. However, the movements were more economical.

VR Trainer is Superior or Inferior to Other Trainers?

Other alternatives to VR trainers are: box trainer, hybrid simulator, augmented reality simulator, porcine model, and pulsating organ perfusion (POP) trainer [5].

VR trainer versus video trainer:

RCT conducted by Hamilton et al. randomized junior surgical residents (n=50) into video trainer (VT) group and VR trainer group (MIST VR). Baseline skill-testing was done for both VR and VT groups. Then they were given training in their respective training groups, and both groups were subjected to post-test in both VT and MIST VR. To assess the correlation of practice in either of the systems with improved OR performance, all the second-year residents (n=19) performed laparoscopic cholecystectomies for symptomatic cholelithiasis before and after the training period and were assessed using GOALS score. In the post-test conducted on MIST VR, the task performance improved significantly in both VT and VR groups. However, the VR group showed more improvement in task performance than the VT group. Similarly, in the post-test conducted on video trainers, the VT group showed more improvement than the VR group. There was a significant overall improvement in the GOALS score of the VR group but no significant improvement in the VT group. This study shows that skills learned on one trainer are transferrable to another and also highlights the fact that VR training is more efficient than VT training [26].

VR trainer versus box trainer:

A study conducted by Brinkmann et al. randomized surgical novices into VR training group and box trainer group. Both were trained for five days and were subjected to a post-test (Laparoscopic cholecystectomy on the porcine gallbladder). Their performance was evaluated using the GOALS score. Both the groups showed improved performance after simulation training. Surprisingly, the box trainer group achieved a significantly higher GOALS score. There is a possibility that the box group performed better because their assessment was in box trainer too. And box training group also had the advantage of training with the same instruments as used in the OR and getting real haptic feedback [27]. However, a meta-analysis conducted by Guedes et al. showed that VR training was better than box training, considering the scores while performing minimally invasive surgery and time to complete (TTC) basic peg transfer task, though no significant difference was observed in TTC of other basic tasks and advanced tasks. Although this meta-analysis had its limitations (publication bias and unblinded reviewers), it widely studied different randomized controlled trials performed in various countries and is more reliable. The author also mentioned in his literature review the views of other authors who opined that VR trainers had better outcomes than box trainers [28]. RCT conducted by Khan et al. showed that skills retained through fundamentals of laparoscopic surgery simulator (FLS) lasted longer than LapSim® VR trainer. The author suggested that VR refresher courses should be conducted shortly after the initial VR training course. Hence, we can summarize that skills are efficiently acquired during VR training but poorly retained as compared to box trainers [29]. But the RCT conducted by Oussi et al. using box trainer and Lap Mentor^TM^ VR trainer showed that the performance of the box training group matched with the VR training group. Hence the author suggests a low-cost box trainer as an effective alternative to the expensive VR trainer [30]. This is also supported by the RCT of Yiasemidou et al., where the box trainer group showed significant improvement in GOALS score compared to the VR trainer group during post-training VR Laparoscopic cholecystectomy post-test in all other metrics except for time taken to complete [31]. The question of the usage of box trainers or VR trainers for laparoscopic training is still debatable and needs further studies considering all other factors.

VR versus low-cost blended learning:

VR being an expensive method of training, the use of multiple affordable alternatives for training was tried. RCT was conducted by Nickel et al. randomized surgical novices into the VR group and blended learning (BL) group. VR group received 12 hours of training using Lap Mentor II and the BL group received 10 hours of basic skills training in box trainer and two hours of e-learning for laparoscopic cholecystectomy training. Then all the participants were subjected to multiple-choice questions (MCQs) post-test for knowledge assessment and POP trainer for assessment of surgical skills. Their performance was rated using the OSATS score. VR group operated faster than BL group and BL group scored higher in MCQ test than VR group. However, the OSATS score was nearly equal for the VR group and BL group. Hence it can be concluded that VR training and BL training are equally effective in training laparoscopic surgeons [32]. RCTs comparing VR trainers and other trainers are summarized in Table 2.

**Table 2:** Summary of RCTs comparing Virtual Reality trainers and other trainers RCT - Randomized Controlled Trial; VT - Video Trainer; GOALS - Global Operative Assessment of Laparoscopic Skills; VR - Virtual Reality; OSATS - Objective Structured Assessment of Technical Skills; HUESAD - Hiroshima university Endoscopie Surgical Assessment Device; MCQ - Multiple Choice Questions; BT - Box Trainer; BL - Blended Learning;  FLS - Fundamentals of Laparoscopic Surgery; MIST - Minimally Invasive Surgical Trainer; MIST VR - Minimally Invasive Surgical Trainer Virtual Reality; POP - Pulsating organ perfusion; FLS - Fundamentals of laparoscopic surgery simulator

Author	Type of study	Objective	Platform(s)	Assessment method	Results
Brinkmann et al. [27]	RCT	Box training or VR training results in better transfer of laparoscopic basic skills into the surgical procedure?	Box trainer, Lap Mentor II, Porcine liver	(GOALS) score	Learning curves were similar to both groups. Box trainer group achieved a higher (GOALS) score
Hamilton et al. [26]	RCT	Comparison of video trainer and VR trainer for improvement of psychomotor skills and surgical laparoscopy procedure	Southwestern centre for minimally invasive surgery guided endoscopic module (SCMIS GEM) video trainer, MIST VR simulator	Global assessment score, scores generated by MIST VR&VT systems	Transfer of psychomotor skills was notably higher in the MIST VR group. Operative scores were also higher in MIST VR group. However, there is no difference between post training scores between both the groups
Khan et al. [29]	RCT	Comparison of maintenance of laparoscopic skills learned using box trainer and virtual reality simulator	(FLS) simulator,LapSim® VR simulator	FLS task-assessment criteria, VR metrics	Initial decay of laparoscopic skills was higher in the VR group than FLS simulator group
Nickel et al. [32]	RCT	Comparison of virtual reality (VR) training with low cost-blended learning (BL)(box + e-learning) of laparoscopic cholecystectomy	E-learning, box trainer, Lap Mentor II, POP trainer	MCQ post-test, (OSATS) score	The VR and BL groups showed equal operative performance in the OSATS score but the VR group performed operation faster. However, the BL group scored significantly higher in the MCQ test concerning LC.
Oussi et al. [30]	RCT	Assessment of trainee performance after laparoscopic training using a box trainer (vs) VR trainer	Black box, Lap Mentor^TM^ VR trainer, MIST VR simulator	MIST VR score	The black box group (particularly females) performed well in the MIST VR post-test than the Lap Mentor^TM^ group
Sumitani et al. [3]	RCT	To determine the optimal order of imparting VR and box training programs to achieve better outcomes	Endowork Pro II box trainer, LapSim® VR trainer, Hiroshima university Endoscopie Surgical Assessment Device (HUESAD)	(HUESAD) skill assessment score	VR Training followed by Box training effectively improved the dexterity of novice surgeons during laparoscopic (combination) training.
Yiasemidou et al. [31]	RCT	Assessment of take-home box trainers (BT) as an alternative to VR trainers	Inovus surgical solutions Box trainer, Lap Mentor^TM^ VR trainer.	GOALS score, VR metrics	BT group improved significantly in VR metrics in post-test compared to VR group. There is no significant improvement in the GOALS score of (BT) group than that of the VR training group.

Barriers to Implementation of VR Training in Surgery

It is an expensive mode of training that totally increases the cost of laparoscopic training, leading to restricted availability. It lacks haptic feedback of tissue. The operative field is visualized on a separate screen away from the patient's axis [33]. VR also has side effects like cybersickness (nausea, vomiting, eye fatigue, dizziness, ataxia), etc. [34]. Another major drawback is using the simulators only for courses and improper maintenance & protection. An effective solution to this is to make them available out of office hours for practice to students under closed-circuit television (CCTV) surveillance instead of locking the doors of the simulation room. VR training program must be a part of the regular surgical laparoscopic training program. Benchmarks to proficiency should be set rather than subjecting them to VR training for a stipulated time. Once these are achieved, they are obliged to provide the operating opportunity to others [35].

Limitations of the study

Most of the studies included in this review are RCTs, and many of them had small sample sizes. The review included literature only from the past ten years (2010-2021). In one of the RCTs taken into consideration, participants felt VR trainers were difficult to use because of the technical difficulties that occurred during the trial. In one of the reviews taken into the study, all the RCTs included in that review had a high risk of bias [13]. Few of the RCTs included in this review did not complete their trial by the time of their publication [16, 24]. There are few dropouts in a few RCTs, which may have affected the study.

## Conclusions

The primary objective of this study is to study the effect of VR in training laparoscopic surgeons and various factors affecting the outcomes of VR training. Laparoscopy is a key procedure that itself poses few challenges to surgeons while operating. Hence it is recommended that laparoscopic procedures need to be simulated and trained before they are performed in the OR. VR can be successfully used for laparoscopic training curriculum as it not only helps to reduce the physical and mental workload on surgeons but also improves their surgical performance in operation theatres. It can also be used to train surgeons to cope up with other technical problems encountered during surgery simultaneously. Competitive training and warmup exercises before VR training can improve few aspects of VR training, but it can be concluded that instructor feedback from mentors, deliberate practice of trainees, and early introduction of haptics in VR can maximize the effect of VR training. This review carefully assessed various factors affecting VR training so that an effective structured VR curriculum can be developed considering all these factors due to the high cost involved in introducing VR into training. Our review also assessed cheaper modalities of simulation like box trainers to see if they are equally effective and can act as a cost-effective replacement to VR trainers. However, we are unable to conclude the same due to variation in results from different studies. This is an area that warrants more amount of research in future studies.
